# Data-dependent and -independent acquisition lipidomics analysis reveals the tissue-dependent effect of metformin on lipid metabolism

**DOI:** 10.21203/rs.3.rs-2444456/v1

**Published:** 2023-01-10

**Authors:** Grace Scheidemantle, Likun Duan, Mareca Lodge, Magdalina J Cummings, Dalton Hilovsky, Eva Pham, Xiaoqiu Wang, Arion Kennedy, Xiaojing Liu

**Affiliations:** North Carolina State University

## Abstract

**Introduction:**

Despite the well-recognized health benefits, the mechanisms and site of action of metformin remains elusive. Metformin-induced global lipidomic changes in plasma of animal models and human subjects have been reported. However, there is a lack of systemic evaluation of metformin-induced lipidomic changes in different tissues. Metformin uptake requires active transporters such as organic cation transporters (OCTs), and hence, it is anticipated that metformin actions are tissue-dependent. In this study, we aim to characterize metformin effects in non-diabetic male mice with a special focus on lipidomics analysis. The findings from this study will help us to better understand the cell-autonomous (direct actions in target cells) or non-cell-autonomous (indirect actions in target cells) mechanisms of metformin and provide insights into the development of more potent yet safe drugs targeting a particular organ instead of systemic metabolism for metabolic regulations without major side effects.

**Objectives:**

To characterize metformin-induced lipidomic alterations in different tissues of non-diabetic male mice and further identify lipids affected by metformin through cell-autonomous or systemic mechanisms based on the correlation between lipid alterations in tissues and the corresponding in-tissue metformin concentrations.

**Methods:**

Lipids were extracted from tissues and plasma of male mice treated with or without metformin in drinking water for 12 days and analyzed using MS/MS scan workflow (hybrid mode) on LC-Orbitrap Exploris 480 mass spectrometer using biologically relevant lipids-containing inclusion list for data-independent acquisition (DIA), named as BRI-DIA workflow followed by data-dependent acquisition (DDA), to maximum the coverage of lipids and minimize the negative effect of stochasticity of precursor selection on experimental consistency and reproducibility.

**Results:**

Lipidomics analysis of 6 mouse tissues and plasma using MS/MS combining BRI-DIA and DDA allowed a systemic evaluation of lipidomic changes induced by metformin in different tissues. We observed that 1) the degrees of lipidomic changes induced by metformin treatment overly correlated with tissue concentrations of metformin; 2) the impact on lysophosphorylcholine and cardiolipins was positively correlated with tissue concentrations of metformin, while neutral lipids such as triglycerides did not correlate with the corresponding tissue metformin concentrations.

**Conclusion:**

The data collected in this study from non-diabetic mice with 12-day metformin treatment suggest that the overall metabolic effect of metformin is positively correlated with tissue concentrations and the effect on individual lipid subclass is via both cell-autonomous mechanisms (cardiolipins and lysoPC) and non-cell-autonomous mechanisms (triglycerides).

## Introduction

Metformin, a biguanide, has health benefits that extend far beyond diabetes. It also has an efficient safety profile, making metformin's mechanisms and site of action an attractive research opportunity^1^. Metformin and other biguanides, such as phenformin, were thought to regulate hepatic lipid metabolism via activating hepatic AMPK, inhibiting de novo lipogenesis, and promoting fatty acid oxidation in liver and muscle^1^. However, emerging evidence tends to support a more complicated mechanism which includes^1-4^: (1) modifications of systemic metabolism such as the reduction of blood glucose and insulin; (2) cell-autonomous effects, which are mediated by the inhibition of mitochondrial metabolism with the alteration of NADH/NAD + ratio and or impairment of intracellular energetic status. However, there is a lack of quantitative measurement of to what extent lipid metabolism in different tissues is directly affected by metformin in vivo. Furthermore, phenformin, a more hydrophobic biguanide with better bioavailability than metformin was withdrawn from the market due to severe toxicity^5^, suggesting that a better understanding of metformin actions will be required to develop potent yet safe drugs.

Unlike phenformin which freely diffuses across a cell membrane, metformin uptake requires active transporters such as organic cation transporters (OCTs), which create asymmetry of free metformin concentrations between tissues and plasma and limit metformin bioavailability^6, 7^. Hence, it is anticipated that the direct actions of metformin may be localized in high OCTs-expressing tissues such as intestine, and the effects of metformin in tissues with low expression of OCTs may be via non-cell-autonomous mechanisms. Due to high expression of OCTs in intestine^8^, metformin concentrations were found to be highest in the intestine^9, 10^, and the intestine has emerged as an important action site of metformin. A recent study reported that metformin’s beneficial effect was attenuated in mice with intestinal epithelium-specific AMPK alpha 1 deletion^11^, suggesting that the action of metformin in intestine is required for correcting metabolic disorders.

It is also unclear whether the mechanism of action of metformin may be dependent on the glycemic state of the individual. The majority of prior studies were focused on diabetic mice, but there are limited studies reporting metabolic effects of metformin in non-diabetic mice. The goal of this study is to systematically and quantitatively investigate metformin-induced metabolic alterations in different tissues of non-diabetic mice. Such a systemic investigation of tissue-dependent metformin effects will advance our understanding of cell-autonomous and non-cell-autonomous effects of metformin. To ensure consistent coverage of lipids detected in different types of tissues, in this study, we chose to prioritize the precursor ions for MS/MS scan by using biologically relevant lipid inclusion list (BRI-DIA) followed by data-dependent acquisition. In our prior study^12^, biologically relevant inclusion list was built to include major lipids present in the prominent database (e.g., LIPID MAPS and or LipidBlast library)^13-15^. These efforts were shown to decrease the stochasticity of precursor selection in MS/MS scan and ensure consistent lipid coverage^12^, which we believe is important for analyzing samples of different origins and matrix. The findings from this study will provide insights into the development of more potent yet safe drugs targeting a particular tissue to combat metabolic syndrome without inducing unwanted consequences.

## Materials And Methods

### Reagents

Metformin hydrocholoride was obtained from TCI America. Metformin-d6 hydrochloride was obtained from Santa Cruz Biotechnology. Optima LC-MS grade of ammonium formate, formic acid, water, acetonitrile, isopropanol, and methanol were purchased from Fisher Scientific. HPLC grade ethyl acetate was purchased from Millipore Sigma. Methyl tert-butyl ether (MTBE) was purchased from Acros Organics. The following lipid standards were obtained from Avanti Polar Lipids: 15:0–18:1(d7) PC, 15:0–18:1(d7) PE, 15:0–18:1(d7) PS, 15:0–18:1(d7) PG, 15:0–18:1(d7) PI, 15:0–18:1-d7-PA, 18:1(d7) LPC, 18:1(d7) LPE, 18:1(d7) Chol Ester, 18:1(d7) MG, 15:0–18:1(d7) DG, 15:0–18:1(d7)-15:0 TG, 18:1(d9) SM, and Cholesterol (d7).

### Animal models

C57BL/6 mice were housed North Carolina State University Biological Resources Facility with ad libitum access to food (Laboratory Rodent Diet 5001) and water on a 12-hour light/dark cycle. At the age of 8 weeks, mice were administrated metformin in their drinking water (1mg/ml) for 12 days. Before tissue and blood collection, mice were fasted for 5 hours (6am-11am). Mice were then anesthetized with isoflurane and sacrificed via cervical dislocation. Blood was collected into tubes containing EDTA anticoagulant via cardiac puncture. Plasma was obtained by centrifugation of whole blood at 1,500 g for 10 min at 4°C and stored at −80°C freezer. Tissues were immediately snap-frozen in liquid nitrogen, except that small intestines were rinsed with PBS and then the jejunum portion was stored in −80°C freezer until further analysis. All animal procedures were approved by the Institutional Animal Care and Use Committee (IACUC) at North Carolina State University.

### Plasma insulin measurements

Insulin in plasma samples was measured using the Rat/Mouse Insulin ELISA Kit (Sigma-Aldrich), following the vendor’s instructions. Briefly speaking, 5 μl sample and 5 μl assay buffer were added to each well. The plate was left to shake with the substrate solution for 30 minutes before stop solution was added. Absorbance was read at 450 nm and 590 nm in a plate reader. Results were analyzed on excel and sample well absorbances were multiplied by the dilution factor of two. Results were graphed using GraphPad Prism.

### RNA Isolation and Analysis

Total RNA was isolated from mouse liver using the Direct-zol RNA MicroPrep Kits (Zymo Research). RNA purity and concentration was determined using a nanodrop. cDNA was made using qScript cDNA SuperMix (Quantabio) and subjected to real-time polymerase chain reaction (RT-PCR) amplification with gene-specific primer using PerfeCTa qPCR FastMix II (Quantabio). The following primers were used for Taqman gene expression assay (Thermo Fisher Scientific): fatty acid synthase (FASN, #Mm00662319_m1); sterol regulatory element binding transcription factor 1-C (SREBP1-C, #Mm00550338_m1); acetyl-CoA carboxylase (ACC1, #Mm01304258_m1), stearoyl-CoA desaturase 1 (SCD1, #Mm00772290_m1), phosphatidate phosphatase (LPIN3, #Mm00499095_m1). Relative abundance of mRNA was normalized to endogenous control 18S.

### Estimation of phospholipase A2 (PLA2) activity in small intestine

Phospholipase A2 Activity Assay Kit (Fluorometric) was purchased from BioVision. To make tissue lysate for PLA2 activity measurement, around 10 mg of mouse jejunum was lysed on ice using sonicator probe for 30 seconds in 200 ul of Tris-HCl buffer (50 mM, pH 7.5) containing Pierce protease and phosphatase inhibitors (Thermo Fisher Scientific), followed by centrifugation at 10,000 x g for 10 min at 4 °C. The supernatant was transferred to a new Eppendorf tube. Samples were diluted to 0.2 mg/ml based on protein concentrations determined using BCA assay. 5 ul of each sample was used for PLA2 activity measurement following the manufacturer’s instructions. To measure the activity of low molecular weight secretory PLA2, tissue lysate were filtered using 30 kDa MWCO spin columns to remove any high molecular PLA2.

### Lipid and polar metabolite extraction from mouse tissues and plasma

All tissue sample was first homogenized in liquid nitrogen and then 10–20 mg tissues were weighed into a new 1.7 ml Eppendorf tube. Ice cold extraction solvent (400 μl 80% methanol/water) and 10 μl metformin-d6 (50 ng/μl in water) was added to each sample. Geno/Grinder homogenizer was used (1500 rpm, 1 to 2 min) to further break down the tissue chunk and form an even suspension. 200 ul supernatant containing polar metabolites was removed after centrifugation at 20,000 g at 4°C for 10 min and transferred to LC vial for polar metabolite analysis using LC-HRMS. The rest samples (200 μl solvent and insoluble pellet) were briefly homogenized using Geno/Grinder again (1500 rpm, 30 sec) to loosen the bottom pellet and better mix with extraction solvent. 480 μl MTBE and 10 μl internal standard solution were added. After rigorous vortexing, 120 μl water was added to initiate phase separation. All samples were centrifuged at 20,000 g at 4°C for 10 min. The supernatant containing lipids was transferred to a new Eppendorf tube and dried using speed vacuum. Dry pellets were stored in −80°C freezer until ready for LC-HRMS analysis.

To extract metabolites and lipids from mouse plasma, 10 μl plasma was mixed with 10 μl water containing internal standards (50 ng/μl metformin-d6, 5 mM [U-^13^C]-glucose and [U-^13^C]-lactate, and 80 μl ice cold methanol was added. After vortex for 1 min, the mixture was centrifuged with a speed of 20,000 g at 4°C for 10 min, and 20 μl was transferred directly to LC vial without solvent evaporation, followed by LC-HRMS analysis (injection volume, 3 μl) of polar metabolites such as glucose and metformin. To the rest samples (80 μl solvent and pellet), 192 μl MTBE and 10 μl lipid internal standard solution were added. After rigorous vortexing, 120 μl water was added to initiate phase separation. All samples were centrifuged at 20,000 g at 4°C for 10 min. The supernatant containing lipids was transferred to a new Eppendorf tube and dried using speed vacuum. The dry pellets of lipids from mouse tissues or plasma were reconstituted into 300 μl sample solvent (isopropanol: ethyl acetate, 1:1, v/v), and 3 μl was injected to LC-HRMS for lipidomics analysis.

### Construction of biologically relevant inclusion list

Using LIPID MAPS and MS-DIAL 4 as the primary sources^13, 14^, we built an inclusion list for MS/MS scan as described previously^12^, and the inclusion list was included in the Supplementary table 1 of our previous publication^12^.

### HPLC method

Lipid analysis was performed using Vanquish UHPLC (Thermo Fisher Scientific). A reversed phase chromatography method with Xbridge BEH C18 column (2.1× 100 mm, Column XP; 130Å; Waters) was used for compound separation at 40°C. Mobile phase A: water:acetonitrile (8:2, v/v) with 0.1% formic acid and 10 mM ammonium formate, and mobile phase B: isopropanol:acetonitrile (9:1, v/v) with 0.1% formic acid and 10 mM ammonium formate. Linear gradient was: 0 min, 40% B; 1.5 min, 40% B; 5.0 min, 85% B; 12.0 min, 97% B; 16.0 min, 97% B; 16.5 min, 40% B; 21.0 min, 40% B. The flow rate was: 0.15 ml/min. The analysis of metabolites in mouse tissues and plasma was performed using Ultimate 3000 UHPLC (Dionex). A hydrophilic interaction chromatography method (HILIC) with an Xbridge amide column (100 x 2.1 mm i.d., 3.5 μm; Waters) was used for compound separation at 25°C. Mobile phase A: water with 5 mM ammonium acetate (pH 6.8), and mobile phase B: 100% acetonitrile. Linear gradient is: 0 min, 85% B; 1.5 min, 85% B; 5.5 min, 35% B; 6.9 min, 35% B; 10.5 min, 35% B; 10.6 min, 10% B; 12.5 min, 10% B; 13.5 min, 85% B; 17.9 min, 85% B; 18 min, 85% B; 20 min, 85% B. For Ultimate 3000 UHPLC, the flow rate is: 0-min, 0.15 ml/min; 6.9–10.5 min, 0.17 ml/min; 10.6–17.9 min, 0.3 ml/min; 18–20 min, 0.15 ml/min.

### Mass Spectrometry

The analysis of metabolites in mouse tissues and plasma was performed on Q Exactive Plus mass spectrometer (Thermo Fisher Scientific), while the analysis of lipids was performed on Orbitrap Exploris 480 mass spectrometer (Thermo Fisher Scientific). Both mass spectrometers were equipped with a HESI probe and operated in the positive/negative switching mode. When Q Exactive Plus mass spectrometer was used, the relevant parameters are as listed: heater temperature, 120°C; sheath gas, 30; auxiliary gas, 10; sweep gas, 3; spray voltage, 3.6 kV for positive mode and 2.5 kV for negative mode; capillary temperature, 320°C; S-lens, 55. The resolution was set at 70,000 (at *m/z* 200). Maximum injection time (max IT) was set at 200 ms and automatic gain control (AGC) was set at 3 × 10^6^. Lipid analysis was performed on Orbitrap Exploris 480 mass spectrometer (Thermo Fisher Scientific). The relevant parameters of Orbitrap Exploris 480 were as listed: vaporizer temperature, 350°C; ion transfer tube temperature, 300°C; sheath gas, 35; auxiliary gas, 7; sweep gas, 1; spray voltage, 3.5 kV for positive mode and 2.5 kV for negative mode; RF-lens (%), 45. The resolution was set at 120,000 (at m/z 200). The scan range was 200 to 1600 (*m/z*). Automatic maximum injection time (max IT) and automatic gain control (AGC) were used. MS/MS scan was acquired using Hybrid mode (BRI-DIA followed by DDA). The inclusion list containing precursor ions in positive and negative ion mode was described in the Supplementary table 1 of our prior publication. The MS/MS condition for positive or negative ion mode was set as follows: precursor isolation window was set at 1 (*m/z*) for all three methods, HCD collision energy was set at 25%, orbitrap resolution of full scan and MS/MS scan was set at 60,000 and 15,000, respectively. Intensity threshold for DDA and DIA MS/MS scan was set at 10,000.

### Data analysis

LC-MS peak extraction and integration were performed using MS-DIAL with default settings. Lipids identified by MS-DIAL were exported and subjected to further processing as described in our prior publication: 1) Mass error was calculated in an excel sheet and ions with mass error larger than 4 ppm were excluded from further analysis; 2) In addition, there were redundant lipid identifications in the result of MS-DIAL analysis. Under our experimental conditions, certain classes of lipids do not fragment well, such as free fatty acids (FA). FA species were detected using amide column and the identification was based on MS1 and the retention time determined by standard compounds when available.

Integrated peak area of lipids was used to calculate the fold change of lipids in different samples. Metabolite and metformin analysis was performed using Sieve (Thermo Fisher Scientific) based on theoretical *m/z* and retention time determined based on standard compounds. Graphs were generated using GraphPad Prism 8 unless otherwise noted.

## Results

### Tissue distribution of metformin and the correlation with metformin-induced lipidomic changes

**Figure 1:**
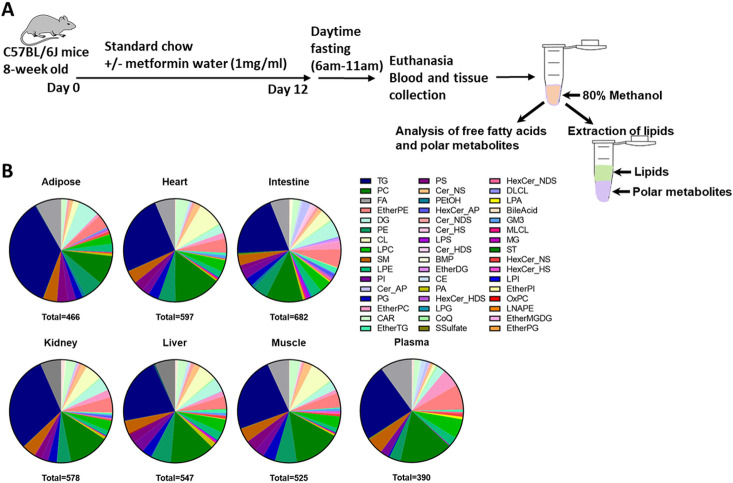
A summary of lipid subclasses measured in various tissues and plasma of male mice. **A.** A schematic diagram of the experimental design. **B.** The number of total lipids and lipid subclasses measured in various mouse tissues and plasma. TG, Triacylglyceride; PC, Phosphatidylcholine; PE, Phosphatidylethanolamine; EtherPE, Ether-linked phosphatidylethanolamine; DG, Diacylglyceride; EtherPC, Ether-linked phosphatidylcholine; SM, Sphingomyelin; LPC, Lysophophatidylcholine; LPE, Lysophosphatidylethanolamine; CL, Cardiolipin; PI, Phosphatidylinositol; Cer_NS, Ceramide non-hydroxyfatty acid-sphingosine; PS, Phosphatidylserine; CAR, Acyl carnitine; PG, Phosphatidylgylcerol; HexCer_NS, Hexosylceramide non-hydroxyfatty acid-sphingosine; PA, Phosphatidic acid; CE, Cholesteryl ester; HexCer_NDS, Hexosylceramide non-hydroxyfatty acid-dihydrosphingosine.

To characterize the short-term effect of metformin on metabolic activities of non-diabetic mice, mice were administered metformin in drinking water (1mg/ml) for 12 days (Fig. 1A). No difference was observed regarding the food consumption and weight gain between control and metformin groups after 12-day treatment (data not shown). To quantitatively evaluate the impact of metformin on different lipid classes in different tissues and plasma, we first checked the coverage of lipid subclasses based on lipid identified in different types of tissues and plasma. The results directly obtained from MS-DIAL tend to contain wrong or redundant lipid identifications, for example, lipids identified with big mass error, redundant identification, wrong retention time, or inadequate fragmentation information. After the removal of wrong and redundant lipid identifications, the numbers of unique lipids identified from different types of samples were: 466 (adipose), 597 (heart), 682 (intestine), 578 (kidney), 547 (liver), 525 (muscle) and 390 (plasma) (Fig. 1B). Lipidomics results exported from MS-DIAL are included in Supplementary table 1. The largest number of cardiolipins was identified in high-metabolic-rate organs such as heart, kidney, liver and muscle, consistent with the role of cardiolipins in supporting oxidative phosphorylation in mitochondria^16^. White adipose tissues are specialized for the storage of energy in the form of triglycerides (TGs), and consistent with this, the largest number of TGs was identified in white adipose tissues. Phosphatidylethanol (PEtOH) is the lipid product of phospholipase D (PLD)-catalyzed reaction between ethanol and PC. PEtOH level increases with the increase of alcohol exposure^17^. PEtOH was found mainly in intestine and adipose tissues, followed by kidney and liver, but not in heart or muscle (Fig. 1B). It is known that gut bacteria such as *E.coli* or yeast convert carbohydrates to aldehyde and then ethanol^18, 19^. Since mice used in this study were not provided with ethanol, endogenous ethanol produced in intestine may lead to the accumulation of PEtOH locally. The PEtOH found in adipose may be due to enzymes (i.e., aldehyde dehydrogenase) responsible for ethanol oxidative metabolism are much lower in adipose tissue than in liver^20^, leading to impotent oxidative metabolism of ethanol in adipose and favoring the formation of non-oxidative metabolism product, PEtOH. Nevertheless, the exact mechanisms warrant further studies.

**Figure 2:**
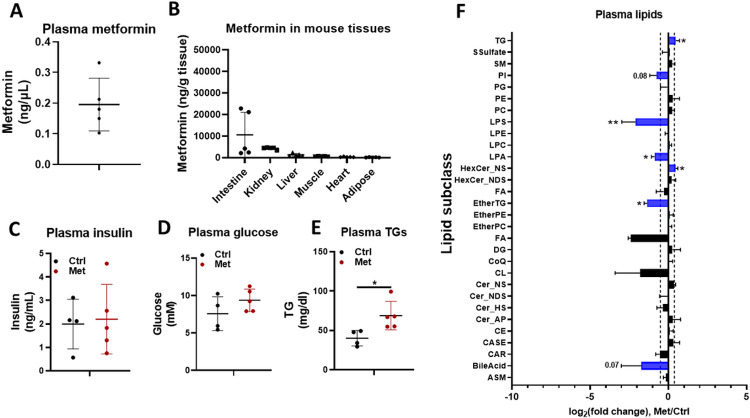
The effect of 12-day metformin treatment on plasma characteristics. **A-B.** The concentrations of metformin in plasma (**A**) and tissues (**B**). The concentrations of insulin (**C**), glucose (**D**), and total TGs (**E**) in plasma of male mice (n=4-5). **F.** The relative changes of the total level of different lipid subclasses in plasma. Values are expressed as mean ± standard derivation. * p<=0.05 and ** p<=0.01 by Student’s two-tailed t test.

Metformin was detected in plasma and various tissues of mice receiving metformin treatment (Fig. 2A). Prior studies demonstrated that metformin is a substrate of organic cation transporters (OCTs), including OCT1, OCT2 and OCT3, and the tissue distribution of metformin involves OCT-dependent cellular uptake of metformin^10^. Consistent with prior findings^10, 21^, our LC-MS results (Fig. 2B) suggested that the concentrations of metformin were highest in small intestine and kidney, which are known to have relatively high expressions of OCTs^8^. Metformin did not induce significant changes of fasting blood glucose and insulin levels (Figs. 2C-D). Metformin unexpectedly increased the triglyceride level in fasting plasma of male mice (Fig. 2E). To characterize the changes of different lipid subclasses in plasma after metformin treatment, we analyzed the fold changes of the total level of each lipid subclass and highlighted lipid subclasses with relatively larger changes (fold change > = 1.3 or < = 0.7, p< = 0.1) (Fig. 2F). These lipid subclasses include TG, EtherTG, LPS, LPA, and HexCer_NS. Several lysoPLs are generated during platelet activation. Plasma LysoPS was suggested as a marker of platelet activation^22^, and hence, a decrease of lysoPS may indicate less platelet activation in mice treated with metformin. The moderate increase of plasma TG by metformin was to our surprise since several studies have shown that metformin decreased plasma TG in mice with hyperglycemia and or hyperlipidemia ^23-25^. A plausible explanation is that mice used in this study are healthy mice and the effect of metformin on plasma TG may be dependent on glycemic status.

### Quantitative characterization of the differential effect of metformin on different lipid subclasses

**Figure 3:**
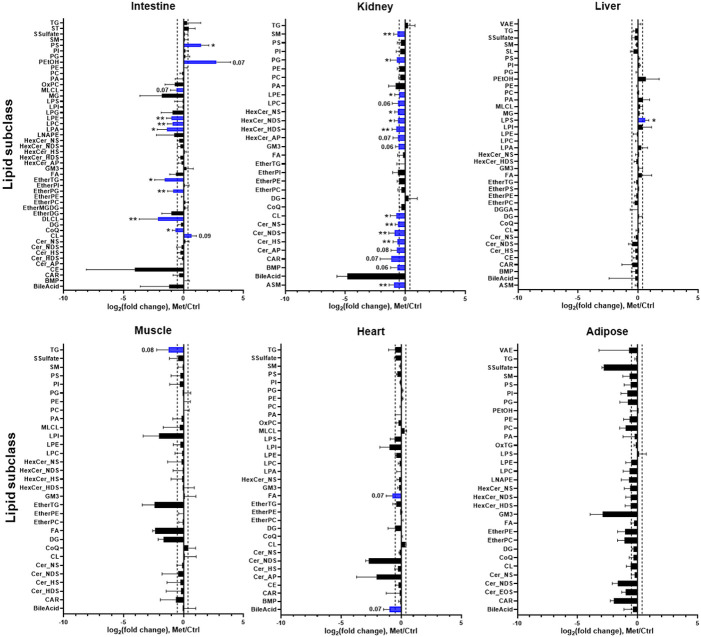
The effect metformin on the total level of different lipid subclasses in different tissues. The total level of each lipid subclass was represented by the sum of peak area of individual lipids in each lipid subclass, and the fold change was calculated by dividing the total level of each lipid subclass in metformin group by the corresponding value in vehicle group. Lipids with relatively larger changes (p<=0.1, fold change >=1.3 or <=0.7) are highlighted in blue and the corresponding significance level or p value is also indicated. Values are presented as mean ± standard derivation. * p<=0.05 and ** p<=0.01 by Student’s two-tailed t test.

To characterize metformin-induced lipidomic changes in different tissues, we analyzed the fold changes of the total level of each lipid subclass in response to metformin treatment (Fig. 3). Metformin induced lipidomic changes to a greater extent in the small intestine (jejunum) and kidney, while in other tissues with relatively lower metformin concentrations, most lipid classes remained unaffected (Fig. 3). In the small intestine (jejunum), PEtOH increased dramatically (fold change = 8.5, p = 0.07) after metformin treatment. The increased PEtOH suggests that metformin may increase endogenous ethanol in the gut. In addition, PEtOH levels in other tissues were not found to be increased by metformin, suggesting that the effect of endogenous ethanol production is localized in the small intestine, which is distinct from the whole-body systemic effect of chronic alcohol consumption. The upregulation of CL may suggest an increase of mitochondrial mass, which may promote mitochondrial oxidative metabolism. The increase of intestinal tricarboxylic acid (TCA) cycle intermediates after metformin treatment (Supplementary Fig. 1) further supports this idea. Dilysocardiolipins (DLCLs), a product of Phospholipase A2 (PLA2)-mediated CL remodeling and degradation, were decreased (fold change = 0.32, p = 0.008) by metformin treatment (Fig. 3). PLA2 enzyme not only hydrolyzes fatty acyl chains in CL to form monolysocardiolipins (MLCLs) or DLCLs, but also hydrolyzed PC at the sn-2 position, releasing free fatty acid and lysophospholipids such as LPC and LPE. The decrease of DLCL, LPC, LPE, and LPA seems to suggest a decrease of PLA2 activity in the small intestine.

**Figure 4:**
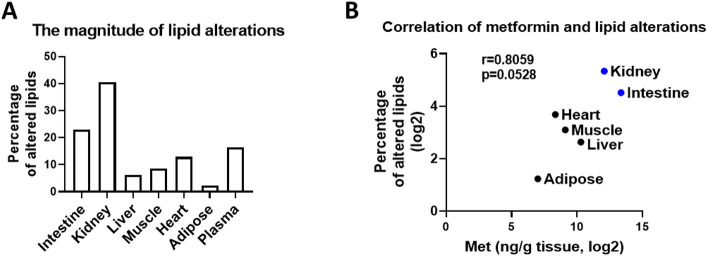
Global lipidomic changes align with in the corresponding concentrations of metformin in different tissues of male mice. **A.** The percentage of altered lipids (p<=0.1, fold change >=1.3 or <=0.7) in mouse tissues. B. The correlation of metformin concentration and percentage of altered lipids in different tissues. The percentage was determined by the number of altered lipids (p<=0.1, fold change >=1.3 or <=0.7) divided by the number of detected lipids in each type of tissues.

**Figure 5:**
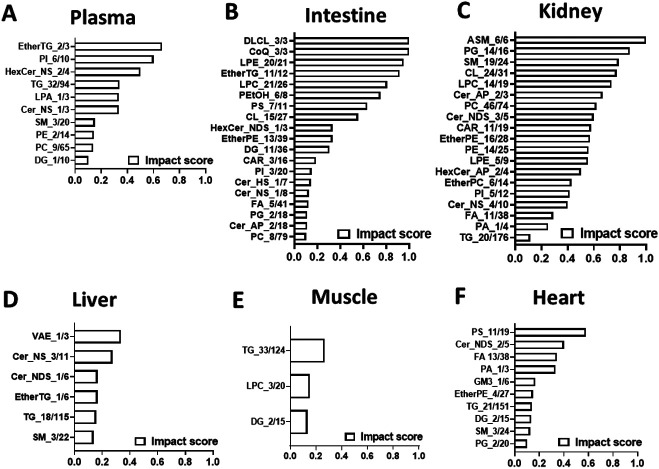
The effect of metformin on lipidome is tissue-dependent. Bar graph of pathway impact score of lipids in plasma (**A**), small intestine (**B**), kidney (**C**), liver (**D**), muscle (**E**), and heart (**F**). Pathway impact score was calculated by dividing the number of altered lipids (p<=0.1, fold change >=1.3 or <=0.7) in each lipid subclass by the number of detected lipids in each lipid subclass.

**Figure 6:**
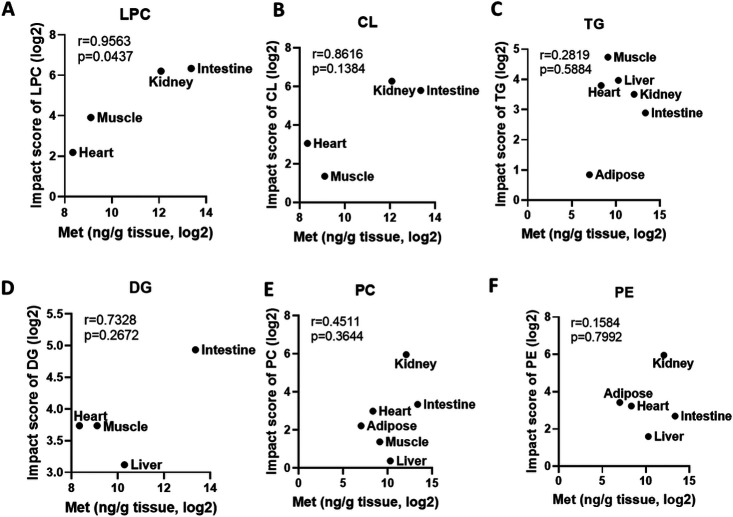
Correlation of alterations of individual lipid subclass with in-tissue metformin concentration in male mice. Correlation of altered LPC (**A**), CL (**B**), TG (**C**), DG (**D**), PC (**E**), and PE (**F**) with metformin concentration in differen tissues. Pathway impact score was calculated by dividing the number of altered lipids (p<=0.1, fold change >=1.3 or <=0.7) in each lipid subclass by the number of detected lipids in each lipid subclass.

To determine whether metformin-induced metabolic alterations are dependent on metformin tissue distribution, we correlated the metabolic changes with metformin tissue concentrations. The results suggest that there is a positive correlation between metformin concentration and the magnitude of metabolic alterations (Fig. 4). To quantitatively evaluate the impact of metformin on different lipid classes, we calculated impact score for each lipid subclass by dividing the number of lipids altered by metformin treatment and the number of lipids detected in each lipid subclass (Fig. 5). The impact score indicates to which extent each lipid subclass was affected by metformin. The results suggest that the impact of metformin on each lipid subclass is tissue-dependent. Metformin treatment altered more than 50% of cardiolipins in organs (small intestine and kidney) with high concentrations of metformin (Figs. 5B-C), but not in other organs with relatively low concentrations of metformin (Figs. 5D-F). In addition, it has been suggested that metformin actions involve both cell-autonomous and non-cell-autonomous effect^1^, and we anticipated that lipidomic alterations via cell-autonomous effects would have a positive correlation with metformin in-tissue concentrations. To quantitatively assess the relationship between the impact of metformin on each lipid subclass and metformin tissue distribution, we calculated the Pearson correlation coefficient between the impact score and the corresponding metformin concentrations. The effect on LPC and CL was modestly correlated with metformin concentrations in tissues (Fig. 6), but for other lipid subclasses. No strong correlation was found between the alterations of neutral lipids such as triglycerides and metformin in-tissue concentrations. These observations suggest that metformin may affect LPC and CL through cell autonomous mechanisms and affect other lipid subclasses via non-autonomous mechanisms.

### Evaluation of lipidomic and metabolic alterations in small intestine of male mice

**Figure 7:**
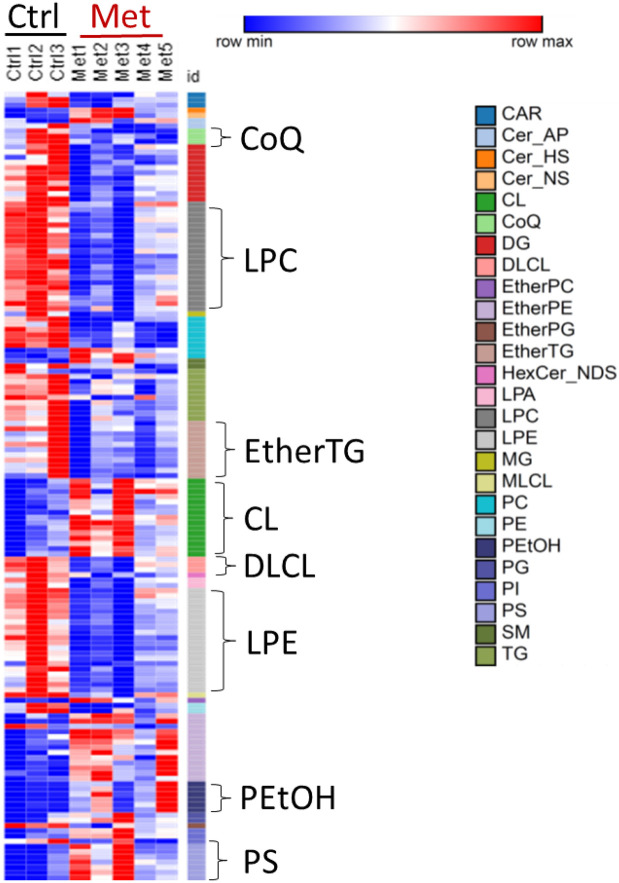
A heat map of altered lipids in male small intestine. Lipids with p<=0.1 and fold change >=1.3 or <=0.7 are included.

**Figure 8:**
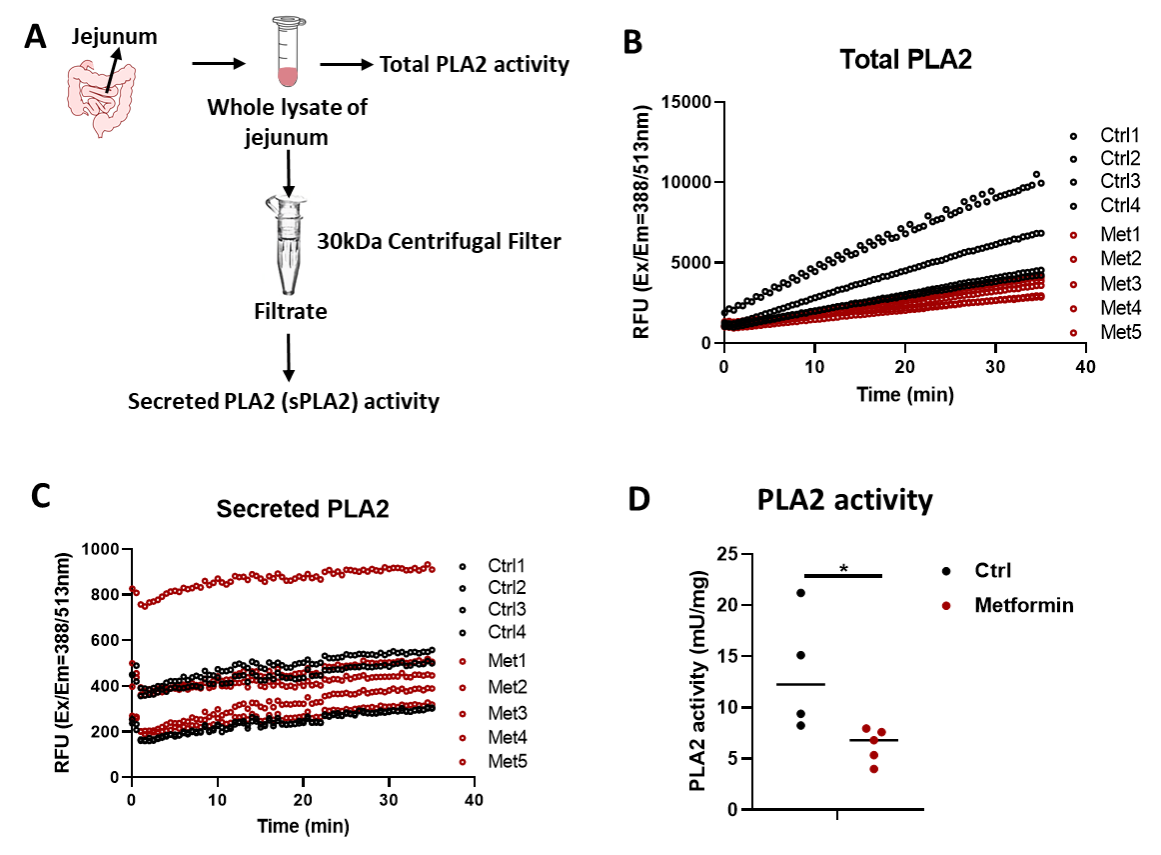
Metformin led to decreased phospholipase A2 (PLA2) activity in small intestine lysate of male mice. **A.** A schematic diagram of the experimental design for testing total and secreted PLA2 activity in jejunum lysate. Total PLA2 activity (**B**) and secreted PLA2 activity (**C**). **D.** The summary of total PLA2 activity in jejunum of male mice. Values are expressed as mean ± standard derivation. * p<=0.05 and ** p<=0.01 by Student’s two-tailed t test.

Given male small intestine was one of the mostly affected organs by metformin treatment, we further investigated individual lipids in lipid subclasses metabolites affected by metformin in the small intestine. As shown in Fig. 7, we highlighted lipid subclasses with an impact score greater than 0.5, and these lipid subclasses include CoQ, LPC, LPE, CL, DLCL, EtherTG, PEtOH, and PS. Consistent with the results shown in Fig. 3, the decrease of individual lipids in DLCL, LPC, LPE, and LPA (Fig. 7) seems to suggest a decrease of PLA2 activity. To test this idea, we measured the enzymatic activity of PLA2 in mouse jejunum lysate by employing a fluorescence (388/513 nm)-based kit, which allows the determination of the total PLA2 and secreted PLA2 activity (Fig. 8). Total PLA2 is composed of cytosolic PLA2 and secreted PLA2. The secreted PLA2 was negligible under our experimental condition, and hence, total PLA2 activity represents cytosolic PLA2 activity. Consistent with the observation of inhibition of PLA2 activity in jejunum lysate, metabolomics analysis of jejunum showed that metabolites such as glycerophosphocholine and phosphorylcholine in glycerophospholipid metabolism were upregulated by metformin treatment (Supplementary Fig. 1). The results suggest that total (or cytosolic) PLA2 activity was significantly lower in the small intestine of mice with metformin treatment. Nevertheless, through which mechanisms metformin affected PLA2 activity warrants further research.

## Discussion

The potential benefits for various diseases and the minimal side effect of metformin attract intensive studies on elucidating the mechanisms and site of action of metformin. The metabolic effect of metformin in vitro and in vivo has been reported in different studies. However, due to the relatively low bioavailability of metformin and the requirement of organic cation transporters for tissue uptake, metformin concentrations in vivo are much lower than the concentrations used in vitro. The pharmacological relevance of metformin concentrations and the related metabolic effects used in in vitro studies remain controversial. To address the issue, we performed a quantitative study to evaluate the tissue-dependent effect of metformin and the correlation with tissue concentrations of metformin. We previously demonstrated that BRI-DIA method using inclusion list comprised of biologically relevant lipids of their optimized ion forms on Orbitrap mass spectrometer improved the consistency of lipid identification, compared to DDA MS/MS scan methods^12^. We used a hybrid approach (BRI-DIA followed by DDA) to characterize metformin-induced lipidomic changes in different tissues and correlated the tissue lipidomic changes with the corresponding metformin tissue concentrations. The results suggest that metformin affects tissue metabolism through both cell-autonomous and systemic mechanisms:

1) The effect of metformin on lysophospholipids and mitochondrial cardiolipins correlates with the intratissue metformin concentrations, suggesting a cell-autonomous effect on these lipid classes. Prior studies suggest that lysophospholipids are naturally occurring surfactants in the small intestine that may damage mucosal cells and epithelial barrier, release lysosomal enzyme activity, and promote inflammation^26,27^. The decrease of intestinal lysophospholipids by metformin may contribute to the anti-inflammation effect of metformin. The observation of metformin affecting mitochondrial lipids only in tissues with high concentrations of metformin is in line with prior evidence that high concentrations of metformin are required to alter mitochondrial metabolism^2, 28-30^, suggesting a localized effect on mitochondrial metabolism.

2) 12-day metformin treatment alters the distribution of triglycerides. In this study, we observed a decrease of intratissue triglycerides in liver and muscle and a moderate increase of plasma triglycerides to 68.84+/−18.11 mg/dl, which is still within the normal plasma triglyceride range (less than 150 mg/dl).The decreased expression of lipogenesis genes (SREBP-1C, FASN, and LIPIN3) in liver suggested the decreased hepatic de novo lipogenesis (Supplementary Fig.2), but it remains to be determined whether it is the consequence of the direct action of metformin in liver or due to the indirect effect of metformin. It was generally thought that metformin directly activates AMPK in liver, and then induces the phosphorylation of ACC, which subsequently inhibits de novo lipogenesis. Nevertheless, evidence also exists that pharmacological relevant dose of metformin did not increase phosphorylation of ACC. In addition, pharmacological or genetic inhibition of de novo lipogenesis was shown to increase fat accumulation in liver. Hence, metformin-induced attenuation of fat accumulation in liver is less likely due to the direct inhibition of de novo lipogenesis. Alternatively, we observed that metformin increased glycolysis in the small intestine, which may subsequently decrease the flow of glucose to liver, which then limits glucose-induced hepatic de novo lipogenesis.

A great body of prior studies reported that metformin decreased plasma triglycerides in mice fed high fat diet^23-25^. It was to our surprise that metformin induced a moderate increase of circulating triglycerides in healthy mice used in this study. It is plausible to speculate that this discrepancy is due to the different health status of mice used in our study and the prior studies. Future studies will be needed to determine whether the current study may be generalized to reflect metformin actions in obese and diabetes.

The decrease of triglycerides in liver and muscle and simultaneous increase of triglycerides in plasma under healthy conditions, suggest a decreased uptake by these tissues. Apolipoprotein A-V (ApoA5) is a protein produced by liver and secreted to blood. It facilitates the lipolysis of circulating triglycerides and tissue uptake of fatty acids by hydrolyzing triglycerides into free fatty acids. Knockdown of hepatic ApoA5 expression decreased plasma triglyceride clearance and reduced tissue lipid uptake^31^. It was reported that metformin decreased ApoA-5, and a decrease of ApoA-5 is expected to increase circulating triglycerides^32^. Future studies will characterize ApoA-5 contributions to fat re-distribution by metformin.

3) In line with the prior discovery of metformin effect on gut microbiota^11, 21, 33^, metformin seems to affect endogenous ethanol production in the small intestine, evidenced by the increase of ethanol and PC product, PEtOH. Even though the exact mechanisms of enhanced ethanol production remain to be elucidated, one plausible explanation is that metformin increases NADH/NAD+ ratio in intestinal epithelial cells, which promotes the conversion of gut bacteria-derived acetaldehyde to ethanol. Nevertheless, this effect is localized in the small intestine only and should be distinguished from the effect of chronic ethanol consumption, which leads to the increase PEtOH in the circulation system and multiple organs such as brain.

This study has a few limitations: 1) The current LC-MS/MS workflow does not determine acyl chain positions or the location of double bonds and hence isomers which differ by locations of acyl chain or double bonds are unresolved. Other separation approaches such as ion mobility or chiral chromatography will be needed for a better separation of these isomeric lipids^34-37^. 2) In this study, n=3-5 per group was used and in future studies, a larger sample size (number of mice per group) would be needed to increase the statistical power. 3) The current analysis of the small intestine (jejunum) samples did not distinguish the different cell types and in future studies, isolation of a particular cell type before lipidomics analysis would be desired to better understand the mechanisms through which metformin affects intestinal lipids.

## Conclusion

The combination of BRI-DIA using inclusion list comprised of biologically relevant lipids and DDA LC-MS/MS data acquisition reduced the redundancy and improved the consistency of lipid identification. Lipidomic analysis of 6 mouse tissues and plasma using this hybrid method suggests that metformin affects tissue metabolism through both cell-autonomous and systemic mechanisms: the metformin effect on mitochondrial metabolism is limited to tissues with high concentrations of metformin while the effect on triglycerides is likely through systemic metabolism. To which extent actions of metformin in small intestine and kidney contribute to the health beneficial effects of metformin will require further studies. In vivo stable isotope tracing assay will be required to quantitatively assess the metformin effect on carbon shuttling in different tissues.

## Abbreviations

LC: Liquid chromatography
HRMS: High-resolution mass spectrometer
DDA: Data-dependent acquisition
DIA: Data-independent acquisition
BRI: Biologically relevant ions
